# Lycium barbarum polysaccharide inhibits ischemia-induced autophagy by promoting the biogenesis of neural stem cells-derived extracellular vesicles to enhance the delivery of miR-133a-3p

**DOI:** 10.1186/s13020-023-00831-8

**Published:** 2023-09-11

**Authors:** Rong Li, Wenjie Duan, Tingle Feng, Chenyang Gu, Qiankun Zhang, Jun Long, Shiying Huang, Lukui Chen

**Affiliations:** 1 Department of Neurosurgery, Neuroscience Center, Integrated Hospital of Traditional Chinese Medicine, Southern Medical University, 13 Shiliugang Rd, Guangzhou, 510310 China; 2 School of Traditional Chinese Medicine, Southern Medical University, Guangzhou, 510310 China

**Keywords:** Neural stem cell, Extracellular vesicles, Lycium barbarum polysaccharide, Autophagy, AMPK/mTOR pathway

## Abstract

**Background:**

Neural stem cell-derived extracellular vesicles (NSC-EVs) mediated endogenous neurogenesis determines a crucial impact on spontaneous recovery after stroke. Here, we checked the influence of Lycium barbarum polysaccharide (LBP) on the biogenesis of NSC-EVs and then focused on studying mechanisms of LBP in ameliorating ischemic stroke outcome.

**Methods:**

LBP was prepared to precondition NSCs and isolate EVs. MCAO models and primary NSCs were administrated to evaluate the therapeutic effect. RT-PCR, western blot, flow cytometry, and immunofluorescence techniques were performed to explore the mechanism.

**Results:**

LBP pretreatment increased the production of NSC-EVs and improved the neuroprotective and recovery effects of NSC-EV in ischemic stroke mice. LBP-pretreated NSC-EV in a dose-dependent manner substantially reduced neuronal death compared with NSC-EV. Screening of the signaling cascade involved in the interaction between NSC-EV and neurons revealed that AMPK/mTOR signaling pathway inhibited autophagic activity in neurons receiving either treatment paradigm. NSC-EVs but not EVs collected from NSCs pretreated with the anti-miR-133a-3p oligonucleotide reduced cell death, whereas the anti-oligonucleotide promoted autophagy activity and cell death by modulating AMPK/mTOR signaling in OGD-induced primary neurons.

**Conclusion:**

LBP activated AMPK/mTOR signaling pathway by increasing the enrichment and transfer of miR-133a-3p in NSC-EVs to inhibit stroke-induced autophagy activity.

## Introduction

Death and long-term disability from stroke cause a heavy burden on global healthcare systems [1]. Ischemic stroke is a process of irreversible neuronal damage and molecular cascade induced by the interruption of oxygen and energy supply secondary to cerebral artery occlusion [2]. Timely intravenous thrombolysis and intravascular thrombectomy are the main treatments for early ischemic stroke, but the short treatment window and strict indications limit their clinical application.

Stem cell intracerebral transplantation to replace the damaged neurons after stroke has a broad prospect for promoting the reconstruction of neural networks. But the harsh microenvironment caused by inflammatory cascade reaction, lipid peroxidation, excitatory amino acid toxicity, etc., leads to a low survival rate (5%) of transplanted stem cells in the brain [3, 4], which limits their application in the acute stage of stroke. Paracrine release of extracellular vesicles (EVs) delivering nutrient factors and molecules is an essential mechanism for stem cells to promote neural repair. The role of EVs in intracellular signal transduction and their therapeutic potential in treating ischemic stroke has recently become a prominent research topic [5, 6].

EVs, as nanosized vesicles (30–1000 nm in diameter), target cell function and mediate intracellular communication by delivering proteins, lipids, and nucleic acids [7]. In multiple clinical and preclinical studies, EVs show low immunogenicity, high engineering plasticity, and a strong ability to penetrate biological barriers. They are becoming a potential therapeutic approach for regenerative medicine [8–10]. In addition, EVs derived from neural stem cells (NSC-EVs) or other stem cell sources appear to be non-inferior to host cells in treating cerebral ischemia [11, 12]. However, the low yields pose a big challenge for the use of such a cell-free therapy originated from NSCs.

In China and Asian countries, herbal medicines such as Lycium barbarum are popularly used in clinics for immunomodulation of various neurological disorders. Lycium barbarum polysaccharide (LBP) is the main bioactive component extracted from Lycium barbarum and has multiple biological effects. LBP alleviates the apoptosis of cortical neurons induced by glutamate [13]. Furthermore, LBP promotes hippocampal neurogenesis in adult rats inhibited by scopolamine [14], suggesting that LBP may promote hippocampal regeneration under pathological conditions. In particular, recent studies demonstrated that LBP could exert functional recovery of motor coordination deficits and neuroprotective effect against mice with cerebral ischemic injury [15–17]. However, the mechanisms of LBP in ameliorating stroke-induced neurological dysfunction have not been fully elucidated. It should be noted that endogenous recruitment of neural progenitor cells is essential for hippocampal neuron regeneration after ischemic brain injury. Therefore, we concentrated on investigating the influence of LBP on NSC and the production of NSC-EVs, which were closely related to neural repair in the present study.

Thus, the current study was designed to determine whether the efficacy of LBP in ameliorating stroke outcomes was based on targeting NSCs to regulate EVs release. We aim to provide scientific evidence for EV production of NSC in the treatment of ischemic stroke and develop a potential agent for stroke therapy.

## Materials and methods

### NSCs isolation and cell culture

Primary NSCs were isolated from pregnant C57BL/6J mice (13–14 days). The cells were cultured in DMEM/F12 supplemented with 2% B27 (Gibco, Grand Island, NY, USA), 20 µg/mL EGF (Sino Biological Inc, Beijing, China), 20 µg/mL bFGF (Novoprotein Scientific, Suzhou, China) in an incubator with 5% CO_2_ at 37 °C. Primary neuron cells were isolated from adult C57BL/6J mice and cultured in DMEM containing 10% fetal bovine serum (FBS) in a CO_2_ incubator. The cell viability was stained with 3-(4, 5 - Dimethylthiazol − 2 - yl) − 2, 5 - diphenyltetrazolium bromide solution (MTT), and the absorbance was detected at 570 nm.

### NSCs pretreated with LBP and their EVs extraction

LBP with a purity > 60% was kindly provided by the Shanghai Institute of Organic Chemistry Chinese Academy of Sciences, China. These compounds were dissolved in deionized H_2_O, and NSCs were administrated with different concentrations of LBP for 72 h. The abbreviations of each group of EV are as follows: NSC-EV: NSCs do not receive any special treatment, and supernatants were harvested after 72 h for EV isolation; L-NSC-EV: the cells were treated with low dose LBP (50 µg / ml); and supernatants were harvested for EV isolation; M-NSC-EV: the cells were treated with moderate dose LBP (100 µg / ml), and supernatants were harvested for EV isolation; H-NSC-EV: the cells were treated with high dose LBP (500 µg / ml), and supernatants were harvested for EV isolation. To determine the role of LBP in mediating NSC-EVs biogenesis, NSCs were incubated with LBP and EV production inhibitor GW4869 for 72 h to evaluate the production.

EVs were extracted using similar procedures as we previously reported [18]. The supernatant of NSCs was centrifuged at 1000 and 3000 g for 10 min, respectively. Next, the supernatant was centrifuged at 10,000 g for 30 min. Then, the collected supernatant was filtered through a 0.22 μm filter. Subsequently, the supernatant was centrifuged at 100,000 g for 90 min. Finally, EV particles were resuspended in PBS and stored at −80 °C.

### Oxygen glucose deprivation and reoxygenation model (OGD/R) and EV treatment

For OGD, the cell culture medium was replaced with glucose-free DMEM and placed in an incubator containing 5% CO2 and 95% N2 at 37 ℃ for three hours. This was followed by subjection to the reoxygenation process under normoxic conditions (21% O2, 5% CO2, and 74% N2) at 37 °C for four hours. To determine the interaction between LBP-pretreated NSC-EV and AMPK, primary neuron cells received a combined treatment of H-NSC-EV (1 × 10^9^/mL) and doxorubicin (AMPK phosphorylation inhibitor, 100 µmol/L) at the start of reoxygenation at 37 °C during the 4 h.

### Transmission electron microscopy (TEM) and nanoparticle tracking analysis (NTA) of size distribution and particle concentration

EVs were observed by transmission electron microscopy (Hitachi H7500, Tokyo, Japan). Samples were fixed with 1% glutaraldehyde, coated on a carbon-coated copper grid, and stained with 1% phosphotungstic acid. The concentration and particle size of EVs were detected by NTA using a ZetaView system (Particle Metrix, Meerbusch, Germany). EVs samples were diluted ten times with PBS to reach optimal concentration for instrument linearity. Size distribution data was generated by applying the Stokes-Einstein equation.

### Animals and middle cerebral artery occlusion model (MCAO)

C57BL/6J male mice (20-22 g) were purchased from Guangdong Medical Laboratory Animal Center (License No. SCXK (Guangdong) 2018-0002). 144 mice were randomly divided into 5 groups: L-NSC-EV group (n = 32), H-NSC-EV group (N = 32), NSC-EV treatment group (n = 32), MCAO group (n = 32) and sham group (n = 16), respectively.

All experimental procedures were performed by institutional guidelines and approved by the Laboratory Animal Ethics Committee of Southern Medical University (Approval No. SMUL2022148). All mice were fasted for 8 h and anesthetized by intraperitoneal injection of 3% sodium pentobarbital (60 mg/kg) before operation. For cerebral ischemia, mice were subjected to MCAO, as described before [17]. The left middle carotid artery was occluded by inserting a 6 − 0 nylon monofilament suture into the left internal carotid artery. Reperfusion was allowed by suture removal one hour after occlusion. Mice in the sham group underwent the identical procedure without suture insertion. Mice with a Longa score of 2–3 were selected for subsequent experiments.

### Labeling, injection, and in vivo tracing of EVs

To monitor peripheral circulating EVs trafficking in the brain, purified EVs were first labeled with fluorescent dye DiR (Umibio Group, Shanghai, China) at the final concentration of 10 mM. EVs (300 µg in 200 µL) derived from NSC after LBP pretreated or PBS was injected into the lateral tail vein of mice 6 h after MCAO, twice per day for 3 days. The Living Imaging System (IVIS Lumina XRNS III, Perkin Elmer, CT, Germany) was used for in vivo visualization of the EV 8 h after injection as instructed.

### Cerebral infarct volume assessment

Mice were anesthetized with intraperitoneal injection of 3% sodium pentobarbital (60 mg/kg) and decapitated seven days after MCAO. The brain tissue was quickly removed, frozen at −20 ℃, and cut into six consecutive coronal sections. The sections were stained with 2% Triphenyl tetrazolium chloride (TTC) solution for 30 min at 37 °C and then fixed in 4% paraformaldehyde solution for 2 h. All stained sections were photographed using a Nikon E 950 digital camera connected to a dissecting microscope, and the infarct area was calculated by Image-J analysis software (Media Cybernetics, Silver Spring, MD, USA). The infarct volume was calculated as the infarct area multiplied by the thickness of the section and expressed as a percentage of the intact contralateral hemisphere volume.

### Laser speckle imaging of cerebral blood flow (CBF)

The mice were anesthetized by intraperitoneal injection of 3% sodium pentobarbital. Then PBS was added to the exposed skull surface, and the cortical blood vessels were visualized by gray focusing. False color threshold and magnification parameters were kept consistent with collecting optical images under Laser Speckle Contrast Imaging System (Rayward Life Technology, Shenzhen, China). The region of interest was selected to obtain binary images to calculate cerebral blood flow.

### Neurological function evaluation

For behavior tests, mice were trained daily for 1 week before MCAO. Tests were conducted on days 3, 7, 14, and 28 post-MCAO.

The grip strength was measured in the forelimbs of each mouse using a Grip Strength Meter (Sans Biotechnology, Jiangsu, China). Each mouse was fixed horizontally, pulled steadily by the root of the tail away from the T bar until its paws left. The peak of the grip strength was measured three times, and the average was used for statistics.

Modified Neurological Severity Score (mNSS) was used to evaluate the neurological function of each group at multiple time points after injury. The scores include motor, sensory, balance beam tests, reflexes, and abnormal movements on an 18-point scale, ranging from 1 to 6 for mild impairment, 7 to 12 for moderate impairment, and 13 to 18 for severe impairment.

The rearing test was employed as described previously [19]. Briefly, forelimb use during explorative activity was analyzed in a transparent cylinder. Preferential single-limb uses, or use of both limbs, were recorded as a percentage of total limb movements while rearing onto the wall and landing. All animals were analyzed for at least 20 exploratory movements along the border and ten landing movements in each session.

### Immunofluorescence staining

Tissue sections or cell slides were blocked in normal donkey serum (1:10, Jackson ImmunoResearch Laboratories, USA) and stained with neuron marker against NeuN (1:500, Abcam, Cambridge, UK) in combination with secondary antibodies conjugated with DyLight 488 (1:200, Abcam, Cambridge, UK). Slides were subsequently washed in PBS and mounted with Fluorescent Mounting Medium containing 4′,6-diamidino-2-phenylindole (DAPI) (ab104135, Abcam, Cambridge, UK). Immunoreactivity was visualized using a fluorescence microscope (AXIO Vert.A1&Imager A2, Carl Zeiss Microscopy GmbH, Germany). ImageJ software (NIH, Bethesda, MD) randomly select five fields under the microscope and compare five views. The number of staining positive cells in the field took the mean of 5 fields to calculate the cell density.

### Neuronal cell death assay

The death rate of neuronal cells was detected by an Apoptosis Detection Kit I (556,547, BD Biosciences, New Jersey, USA). Each group’s primary neuron cells were harvested and made into single-cell suspension, followed by a rinse step with PBS twice. Next, the cells were resuspended in a bending buffer. FITC annexin V and PI were added and incubated with the cell suspension (2 × 10^5^ cells) for 15 min before flow cytometry analysis. Data were collected using a FACSCanto II flow cytometer and processed by FlowJo software.

The cell apoptosis of brain tissue was detected by terminal deoxynucleotidyl transferase dUTP nick end labeling (TUNEL) apoptosis assay kit (Beyotime Biotechnology, Jiangsu, China) according to the manufacturer’s instructions. In brief, frozen slides were incubated with 100 µg/mL proteinase K for 25 min at 37 °C and then incubated with the TUNEL reaction mixture at 37 °C for 2 h. Images were collected by Fluorescent Microscopy. The ratio of TUNEL-positive cells to total neuronal cells indicates the apoptosis index. Images were captured using a Leica DM6B (Leica Microsystems, Milan, Italy). Images were processed with ImageJ software.

### Western blot assay

Total proteins were extracted from NSC-EV or HT-22 cells or mice brain tissues using RIPA lysis buffer (Beyotime Biotechnology, Jiangsu, China) and quantified with the BCA kit (Beyotime Biotechnology, Jiangsu, China). An equal volume of protein was transferred to polyvinylidene difluoride membranes by SDS-PAGE. After blocking with 5% Bovine Serum Albumin in PBS for one hour at room temperature, the membrane was incubated overnight at 4 °C with the corresponding primary antibody including CD9 (1:2000; Abcam, Cambridge, UK), CD81(1:1000; Abcam, Cambridge, UK), Alix (1:1000; Abcam, Cambridge, UK), P62 (1:1000, Cell Signaling Technology Inc, MA, USA), Beclin-1 (1:200, Santa Cruz Biotechnology, CA, USA), LC3-II/I (1:300, Cell Signaling Technology Inc, MA, USA), and AMPK (1:1000; Santa Cruz Biotechnology, CA, USA), p-AMPK (1:1000, Cell Signaling Technology Inc, MA, USA), mTOR (1:1000, Cell Signaling Technology Inc, MA, USA) and p-mTOR (1:1000, Cell Signaling Technology Inc, MA, USA). Next, it was incubated with horseradish peroxidase-conjugated secondary antibodies at room temperature for two hours. Perform ECL as described by the manufacturer and acquire images using the chemiluminescence method.

### Quantitative reverse transcriptase polymerase chain reaction(RT-PCR)

Total RNA was extracted using TRIzol reagent (15,596,026, Invitrogen, USA). Reverse transcription of total miRNA was performed using a miScript reverse transcription kit (205,311, Qiagen, Hilden, Germany) per manufacturer’s protocol. QuantiNova SYBR Green PCR Kit (208,057, Qiagen, Hilden, Germany) and miR-133a-3p and miR-206 specific primers were used to determine the expression of mature miRNAs. RNU6B was used as an internal control.

To detect mRNA expression, cDNA was synthesized using a reverse transcription kit (D7168M, Beyotime Biotechnology, Jiangsu, China) followed by real-time quantitative polymerase chain reaction on an Opticon 2 Real-Time PCR assay system. The primers used were: TNF-α: ATGTCTCAGCCTCTTCTCATTC (forward); GCTTGTCACTCGAATTTTGAGA (reverse). IL-1β: TGAATTCTACTTCCTCACCCAC (forward); GGTACATGCTGTTCTCAAACTG (reverse). IL-6: CTCCCAACAGACCTGTCTATAC (forward); CCATTGCACAACTCTTTTCTCA (reverse). β-actin: CGTCCGTGACATCAAGGAGAAGC (forward); ACCGAGGAAGGAAGGCTGGAAG (reverse). Gene expression at the mRNA level was normalized to β-actin. The relative mRNA expression of target genes was calculated by the 2 ^−△△^Ct method.

### Statistical analysis

SPSS.22 and GraphPad Prism 9.0 software were used for data analysis and processing. For data with normal distribution and homogeneity of variance, the independent t-test was used to compare the mean values of the two groups; One-way analysis of variance (ANOVA) was used to compare the mean values of multiple groups. Between-group differences at multiple time points were assessed using a two-way ANOVA test followed by the Bonferroni post-hoc tests (two-tailed). Kruskal-Walli’s test was used for data that did not conform to the normal distribution, followed by the Games-Howell test for multiple comparisons. Data are expressed as mean ± SD. P < 0.05 was considered statistically significant.

## Results

### Effects of LBP intervention on the characterization of NSC-EVs

**Fig. 1:**
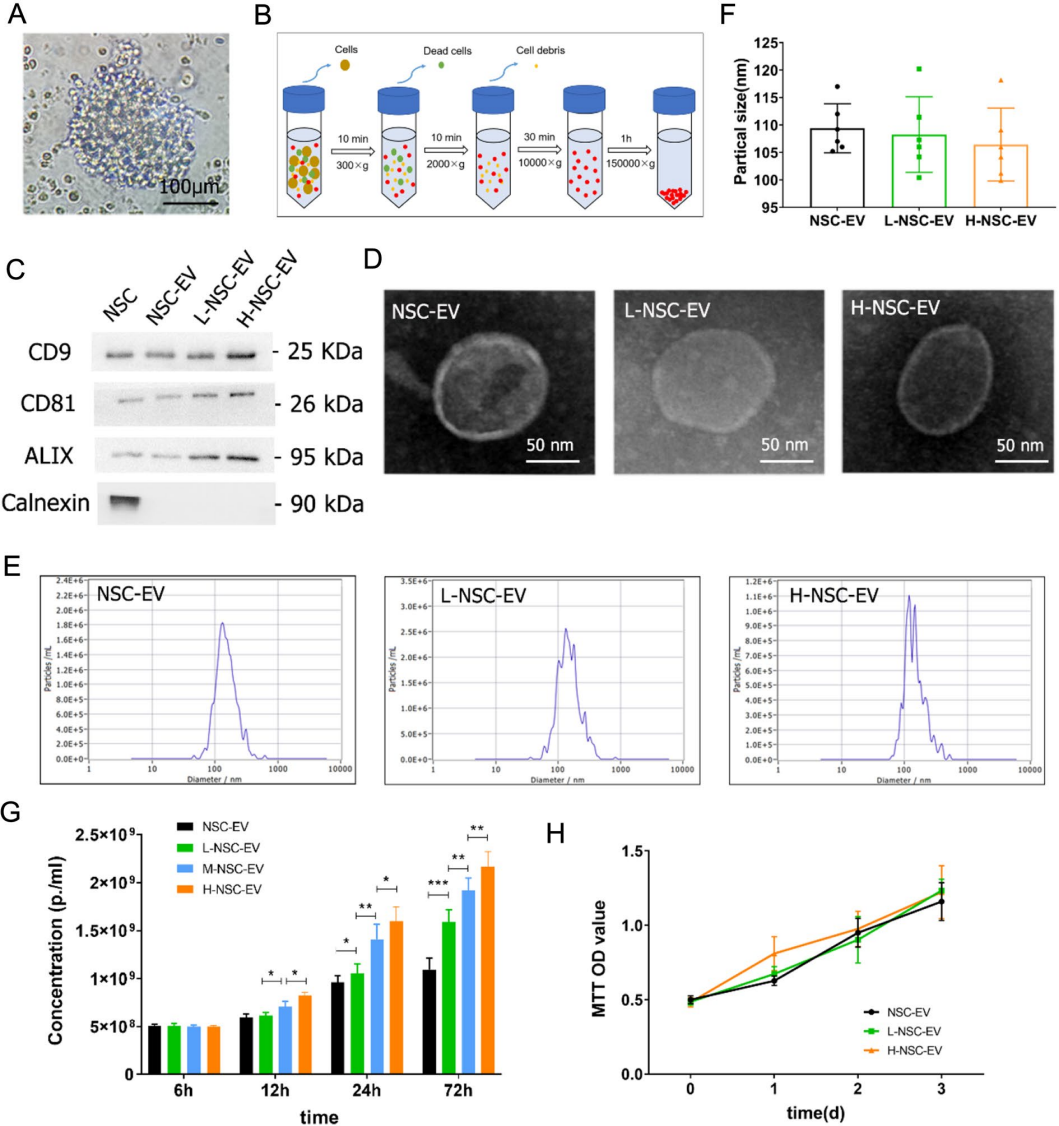
Characterization of EVs derived from NSCs with LBP pretreatment . **A** The cultured primary NSCs showed a typically suspended neurosphere under the optical microscope. **B** A Schematic diagram of sequential ultracentrifugation procedures was applied for EV extraction. **C** Samples of EVs collected from NSC with or without LBP pretreatment were analyzed by Western blot with antibodies against EV-specific markers CD9, CD81, and ALIX. **D** A representative electron micrograph image showing the typical morphology and size range of membrane particles between NSC-EV, L-NSC-EV, and H-NSC-EV groups. **E** NTA was used to determine the concentration and size distribution of EVs between three groups, 24 and 72 h after LBP treatment. **F** Quantification of the particle size and **G** concentration of EVs determined by NTA. **H** Cell viability was analyzed in NSCs exposed to low or high-dose LBP at 1, 2, and 3 days using the MTT assay. Data are shown as mean ± SD. Data are statistically different from each other with *P < 0.05, **P < 0.01, and ***P < 0.001

To investigate whether LBP treatment influences NSCs and their derived EVs. The primary NSCs were isolated and cultured, which showed typical neurospheres (Fig. 1A). Then, EVs were extracted from NSC supernatants by ultracentrifugation (Fig. 1B). Consistent with previous reports [20], western blot analysis showed that CD9, CD81, and Alix were abundantly expressed in these NSC-EVs, and calnexin was undetected (Fig. 1C). No significant morphological differences were identified by TEM analysis between NSC-EVs and NSC-EVs pretreated with LBP, as both showed typical circular vesicles with a diameter of approximately 100 nm (Fig. 1D). We further used NTA to quantify the number and size distribution of EVs, which showed strong enrichment in particles in the range of 50 to 130 nm (Fig. 1E). Interestingly, particle size was not significantly different between groups (109.23 ± 4.62 nm for NSC-EVs, 108.25 ± 6.88 nm for L-NSC-EVs, 106.43 ± 6.64 nm for H-NSC-EVs, Fig. 1F), but a higher concentration of EVs was presented after 24 h of LBP treatment as compared to that after PBS treatment based on the same collected supernatant (Fig. 1G). Moreover, LBP promoted EV biogenesis in a dose-dependent manner, while the yield increased with a time trend. Specifically, the concentration increased more significantly than PBS treatment after 72 h of treatment with 500 ug/ml LBP (1.08 ± 0.11 × 10^9^ particles/mL for NSC-EV; 1.58 ± 0.15 × 10^9^ particles/mL for L-NSC-EV; 1.91 ± 0.18 × 10^9^ particles/mL for M-NSC-EV; 2.17 ± 0.22 × 10^9^ particles/mL for H-NSC-EV, Fig. 1G). Notably, increased EVs release did not alter the viability of NSCs (Fig. 1H), which suggested that LBP stimulation promotes the paracrine release of EVs from NSC rather than affecting the proliferation of NSC.

### LBP pretreated NSC-EVs induce sustained neuroprotection and attenuate ischemia stroke outcome

**Fig. 2:**
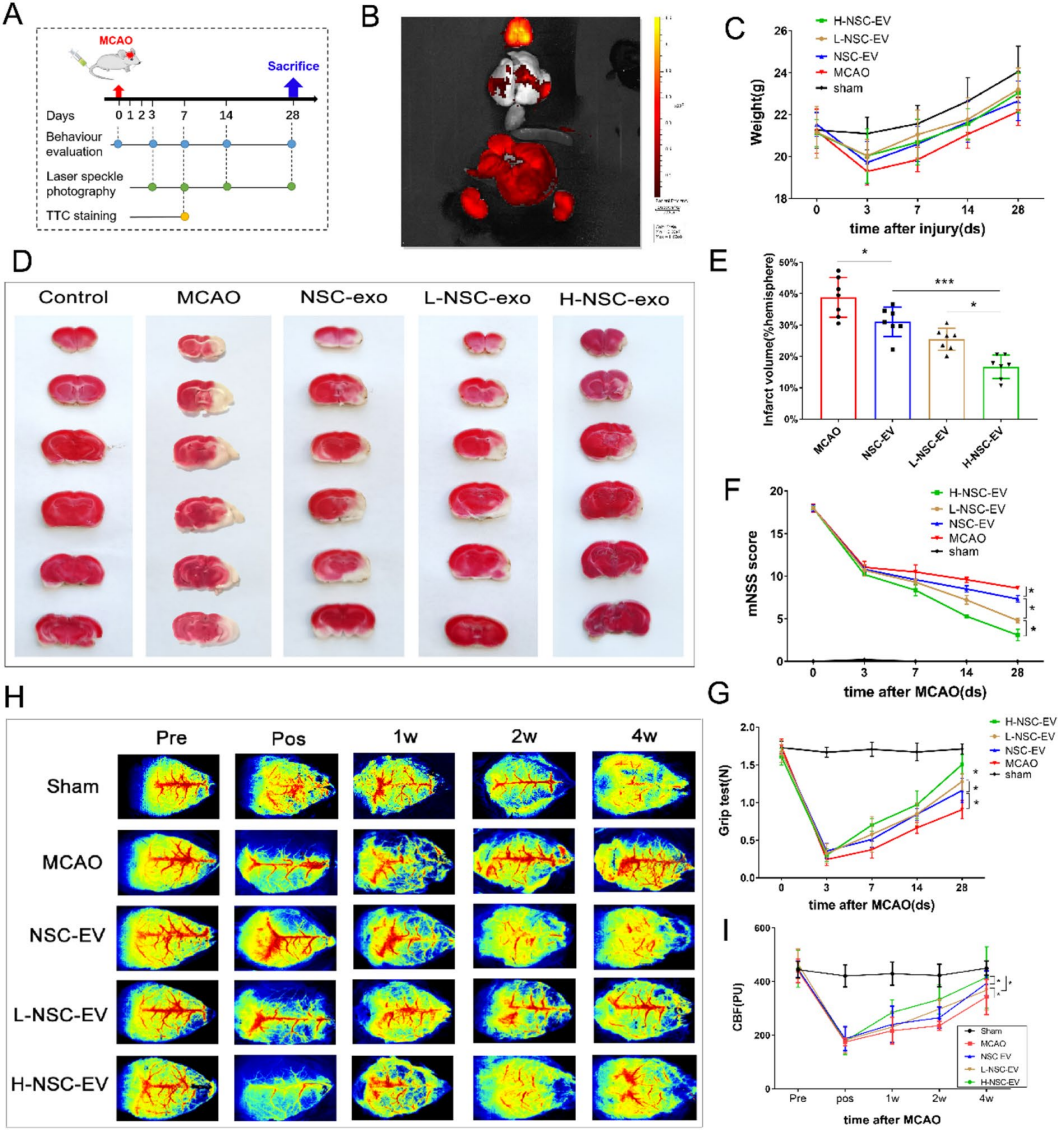
Effects of LBP pretreated EVs on stroke outcome are dose-dependent . **A** In vivo experiment illustration. **B** In vivo imaging of DIR-labeled EV. **C** Changes in body weight of mice in each group throughout the experiment. **D** Representative images of TTC stained coronal brain sections and **E** quantitative analysis of infarct volume of PBS-treated, NSC-EV treated, and L-NSC-EV treated and H-NSC-EV treated rats 72 h after ischemia. **F** and **G** The long-term effect of NSC-EV pretreated with or without LBP on sensorimotor function was assessed by modified mNSS score **F** and grip test **G** 3, 7, 14, and 28 days after MCAO. **H** Representative images and **I** quantification of CBF before, post, and up to 4 weeks after stroke. n = 6 animals per group. Data are shown as mean ± SD. Data are statistically different from each other with *P < 0.05, **P < 0.01, and ***P < 0.001

We herein further investigated whether or not the aforementioned LBP-promoted paracrine effect improves the therapeutic ability of NSC-EVs in stroke. Neurological function assessment, cerebral blood flow monitoring, and infarct volume assessment were performed at different time points, as shown in Fig. 2A. We first checked the EV biodistribution patterns under ischemic conditions. DiR-labeled NSC-EV were injected intravenously into mice immediately at the beginning of the reperfusion. In vivo tracing imaging 8 h after injection showed that most EVs were targeted to the brain across the blood brain barrier (BBB) and a few in the liver and kidney region (Fig. 2B). No significant differences were observed in body weight between groups over time (Fig. 2C). Mice that received LBP-pretreated NSC-EVs exhibited significantly smaller infarct volumes seven days after MCAO compared to NSC-EVs treatment in a dose-dependent manner (Fig. 2D–E). Along with such a reduction of acute brain injury, the behavioral test analysis revealed better neurological improvement in mice receiving LBP-pretreated EVs at all time points. The behavioral evaluation showed that mice receiving LBP pre-treated NSC-EV showed more neurological function at all time points (Fig. 2F–G). Notably, this better test performance in both the grip and mNSS score evaluation remained long-lasting and stable until the end of the observation period of 4 weeks. Likewise, we continued to observe the changes in CBF in the infarcted cortex throughout the experimental period. Consistent with the reduction of neurological impairment, increased CBF was found in mice administrated with LBP-pretreated NSC-EVs compared with animals treated with NSC-EVs over time (Fig. 2H–I). Conclusively, LBP pretreatment improves the therapeutic effect of NSC-EVs on both the histological, imagological, and functional levels in experimental stroke models.

### LBP enhanced the neuroprotective effect of NSC-EVs by inhibiting autophagy

**Fig. 3:**
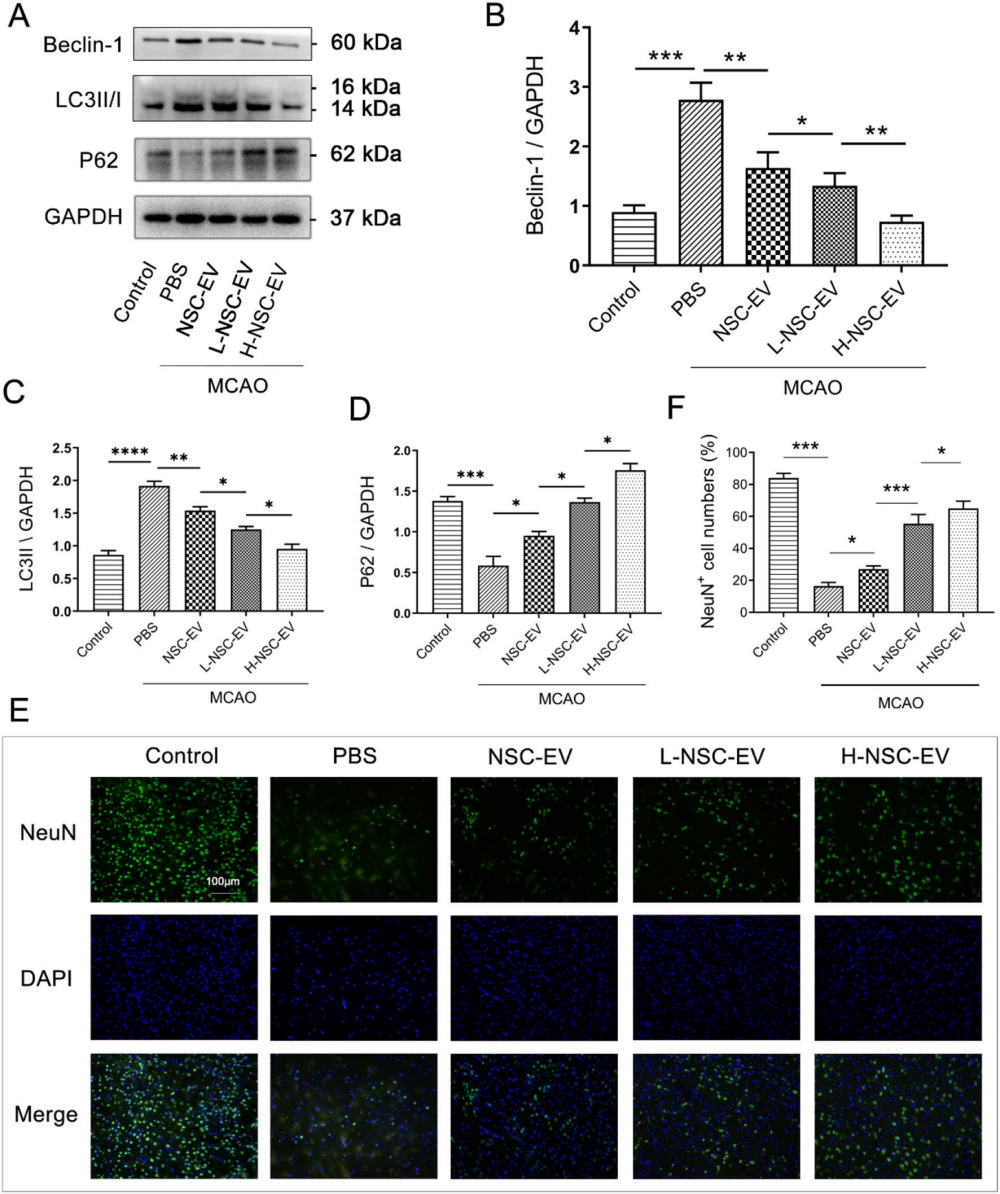
Autophagy regulation induced by LBP pretreated EVs reduces post-stroke brain injury . **A** Western blot analysis of Beclin-1, P62, and LC3II proteins in ischemic brain tissue under PBS-treated, NSC-EV treated, and L-NSC-EV treated and H-NSC-EV treated rats. **B**–**C** The expression levels of selected proteins detected by Western blot were normalized to the GADPH level of the same sample. **E** Representative immunofluorescence images between each group as indicated by NeuN staining within the ischemic lesion site. **F** The quantitative analysis of the cortical neuronal density in ischemia-induced mice at 4 weeks after MCAO. Data are shown as mean ± SD. n = 6 animals per group. Data are statistically different from each other with *P < 0.05, **P < 0.01, and ***P < 0.001

Appropriate autophagy is considered to reduce cell death and the degree of brain tissue damage after stroke [21]. Although recent evidence suggests that autophagy is involved in hypoxia or ischemia-induced cell injury, published data appears contradictory regarding autophagy being beneficial or detrimental [22–24]. We, therefore, examined the expression levels of autophagy-associated proteins to verify the effect of NSC-EVs on neural autophagy activation. When mice were subjected to MCAO and treated with NSC-EVs or LBP-pretreated NSC-EVs, the stroke-induced increased protein abundance of LC3-II and Beclin-1 was reversed, accompanied by a decrease in P62 protein accumulation (Fig. 3A-D). More importantly, LBP-pretreated NSC-EVs further repressed the LC3-II expression and rescued cell injury than NSC-EVs in a dose-dependent manner. In addition, the cell survival rate of cortical neurons was measured after stroke after NSC-EVs treatment. We found that treatment of cortical neurons exposed to MCAO with NSC-EVs or EVs pretreated with LBP yielded a significant reduction of cell injury compared to PBS treatment (Fig. 3E–F). Interestingly, High-dose LBP-pre-treated EVs treatment is superior to NSC-EVs treatment in survival rates of cortical neurons exposed to MCAO (Fig. 3E–F). Combined with the abovementioned results, we showed that LBP pretreatment can promote NSC-EVs to inhibit autophagy flux and protect cortical neurons from ischemic injury in a dose-dependent manner.

### LBP pre-treated NSC-EVs suppressed inflammatory response and reduced neuronal apoptosis after stroke

**Fig. 4:**
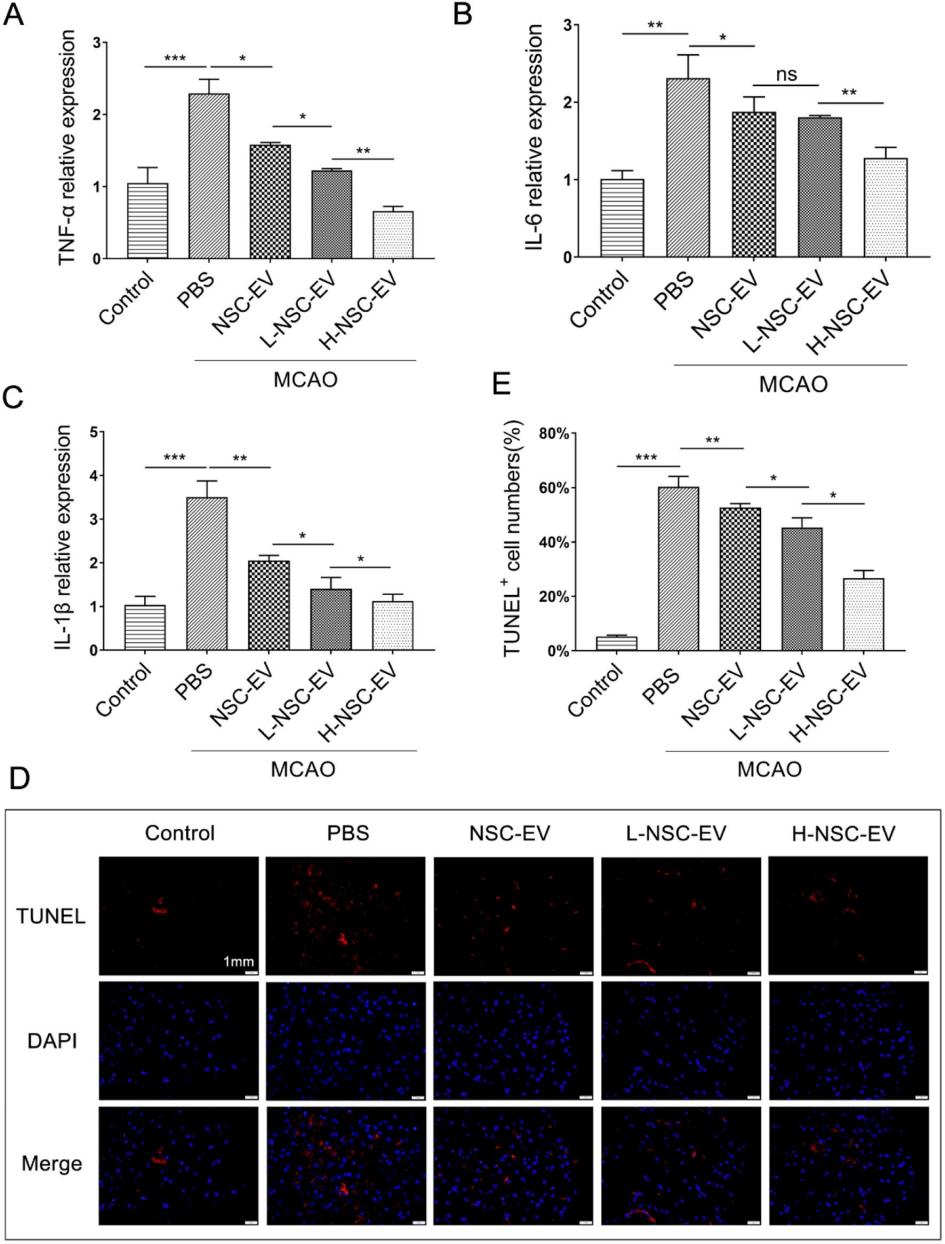
LBP pretreated NSC-EVs suppressed inflammatory response and reduced neuronal apoptosis after stroke . OGD-exposed neurons were incubated with PBS, NSC-EV, L-NSC-EV, and H-NSC-EV. Cells incubated under standard cell culture conditions were used as negative control. **A**–**C** The mRNA expression of TNFα, IL-6, and IL-1β in the sham and ipsilateral side of the infarcted brain was measured by RT-qPCR at post stroke day 3 **D** Representative images and **E** quantification of TUNEL^+^ cells in the infarct border. Data are shown as mean ± SD. Data are statistically different from each other with *P < 0.05, **P < 0.01, and ***P < 0.001

To further evaluate the role of EVs in inhibiting inflammation, RT-PCR and TUNEL staining were applied to evaluate the level of inflammation and neuronal death in brain tissues. The results showed that NSC-EVs treatment decreased the expression levels of pro-inflammatory cytokines IL-1β, IL-6, and TNF-α in MCAO mice (Fig. 4A–C). Compared with NSC-EVs, LBP-treated NSC-EVs further inhibited inflammatory response, which reduced the mRNA expression levels of IL-1, IL-1β, and TNF-α, especially in the H-NSC-EV group (Fig. 4A–C). Unsurprisingly, we confirmed that LBP-pretreated NSC-EVs further dose-dependently reduced the apoptosis rate of neural cells compared with NSC-EVs treatment (Fig. 4D–E). These results suggested that LBP improves the anti-inflammatory and anti-apoptotic effects of NSC-EVs.

### NSC-EV pretreated with LBP reduced autophagic flux through AMPK/mTOR pathway

**Fig. 5:**
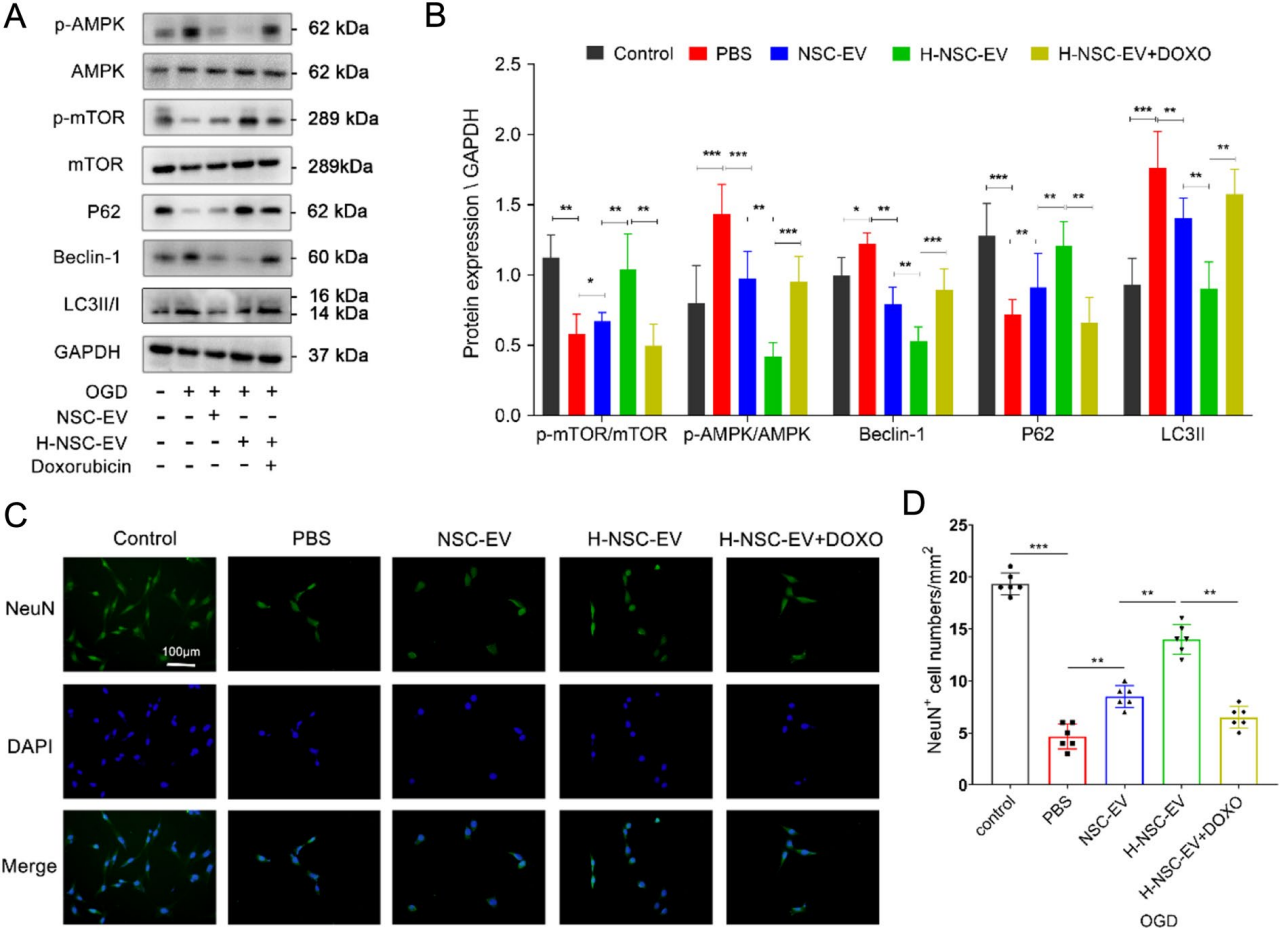
NSC-EVs pretreated with LBP inhibit autophagic flux and protect primary neurons from OGD injury through AMPK/mTOR signalling. OGD-exposed neurons were either incubated with PBS, NSC-derived EVs, or NSC-EV pre-treated with low or high doses of LBP. All experimental conditions were performed with or without the AMPK inhibitor Doxorubicin (Doxo). Cells incubated under standard cell culture conditions were used as negative control. **A**–**B** p-AMPK, p-AMPK, LC3II, Beclin-1, and P62 levels were detected by Western blotting followed by densitometric analysis. **C**-**D** Fluorescent images and quantitative analysis of NeuN^+^ cells exposed to OGD in the aforementioned groups (control, NSC-EV, H-NSC-EV, and H-NSCEV + Doxo). Data are given as mean ± SD. Data are statistically different from each other with *P < 0.05, **P < 0.01, and ***P < 0.001

To reveal the mechanisms by which NSC-derived EVs regulate autophagy, we investigated the autophagy-related signaling pathway. The mTOR pathway is the primary pathway by which mammals regulate starvation-induced autophagy. Accumulating evidence indicates that activation of AMPK inhibits mTOR activity to induce autophagy after acute stroke [25, 26]. Therefore, we in vitro verified whether or not NSC-EVs reduce mTOR-mediated neuronal autophagy by inhibiting AMPK phosphorylation. Indeed, up-regulation of AMPK phosphorylation and down-regulation of mTOR phosphorylation were found in OGD-exposed primary neurons compared to standard cell culture conditions (Fig. 5A–B). Incubation of neurons with either NSC-EVs or with NSC-EVs pretreated with high dose LBP significantly reversed the OGD-induced up-regulation of AMPK phosphorylation and down-regulation of mTOR phosphorylation, with subsequent decreased expression of LC3II and Beclin-1 as well as increased accumulation of P62 (Fig. 5A–B). Furthermore, to reveal the relationship between NSC-EVs and the AMPK pathway, the AMPK phosphorylation inhibitor doxorubicin was applied in primary neurons exposed to OGD. The combined treatment of neurons with doxorubicin and LBP-pretreated NSC-EVs resulted in a reversal of the former EV-induced down-regulation of AMPK phosphorylation and up-regulation of mTOR phosphorylation, which indicated that NSC-EVs act in an opposite way than autophagy through AMPK/mTOR pathway (Fig. 5A–B). Similarly, inhibition of AMPK phosphorylation-induced autophagy reduction with doxorubicin significantly improved neuron viability (Fig. 5C–D), which suggested that a moderate reduction in autophagy regulated by LBP-pretreated NSC-EVs further confers significant protection against OGD-induced cell death in primary neurons.

### Inhibition of EV biogenesis reverses NSC-EV-induced protective effects in neurons exposed to OGD

**Fig. 6:**
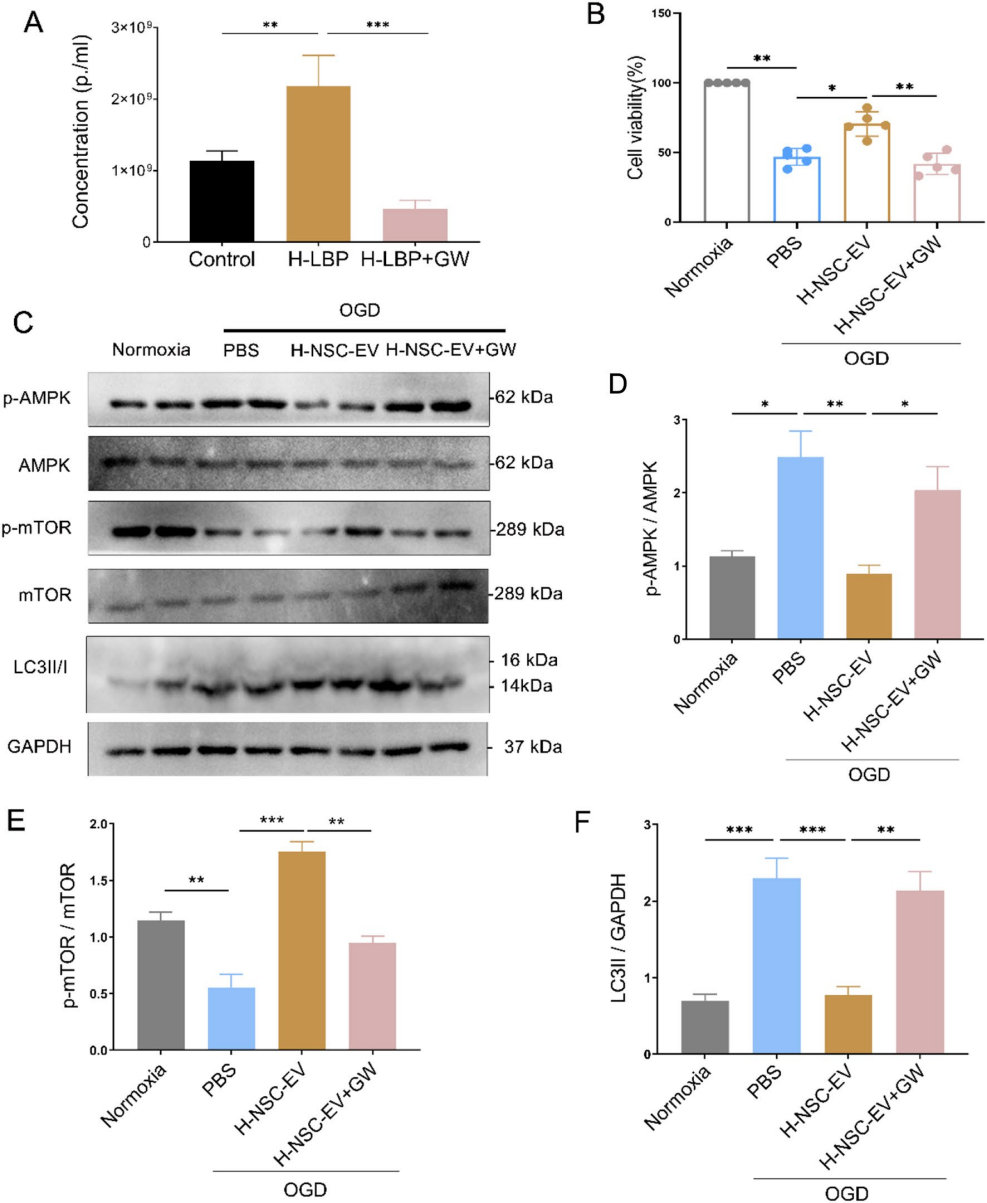
The regulation of the autophagic flux by NSC-EVs depends on exosomes . **A** Numbers of NSC-derived EVs obtained from NSCs pre-treated with PBS, high dose of LBP(H-LBP-EV), and high dose of LBP followed by treatment with the exosome secretion inhibitor GW4869 (H-NSC-EV + GW) were quantified by NTA. **B** Cell viability was examined in OGD-exposed primary neurons treated with PBS, NSC-EV, H-LBP -EV, and H-NSC-EV + GW. Cells incubated under standard cell culture conditions (Normoxia) were defined as 100% cell survival. **C** p-AMPK, p-mTOR, and LC3II were quantified by Western blotting in OGD-exposed primary neurons co-incubated with PBS, H-NSC-EV, and H-NSC-EV + GW. (**D–F **Quantitative analysis of p-AMPK, p-mTOR, and LC3II is shown. Data are presented as mean ± SD. Data are statistically different from each other with *P < 0.05, **P < 0.01, and ***P < 0.001

As we know, exosomes constitute an important subgroup of EVs population. Ceramide synthesis is an essential mechanism for exosome secretion. To determine the role of exosomes in regulating autophagy and neuroprotection in primary neurons exposed to OGD, we inhibited ceramide synthesis with the neutral sphingomyelinase-targeting inhibitor GW4869. As expected, GW4869 pre-treatment led to a significant decrease in exosome secretion shown by NTA analysis (Fig. 6A). Then, we applied GW4869 in the coculture model, and the effects of NSC-EVs promoted by LBP pretreatment on neuroprotection and autophagy regulation were significantly reversed when NSCs were pretreated with the inhibitor of exosome secretion GW4869 (Fig. 6B–D).

Notably, the incubation with NSC-EVs up-regulated the abundance of p-AMPK and p-mTOR in neurons exposed to OGD, while incubation of neurons with EVs collected from GW4869 pretreated NSCs failed to inhibit p-AMPK and p-AMPK protein levels (Fig. 6C, E–F). Hence, exosomes forming a subgroup of NSC-EVs are crucial in mediating the anti-autophagic activity of NSCs in primary neurons exposed to OGD.

### LBP-mediated enrichment of miR-133a-3p in NSC-EVs was essential for enhancing neuroprotection after stroke

**Fig. 7:**
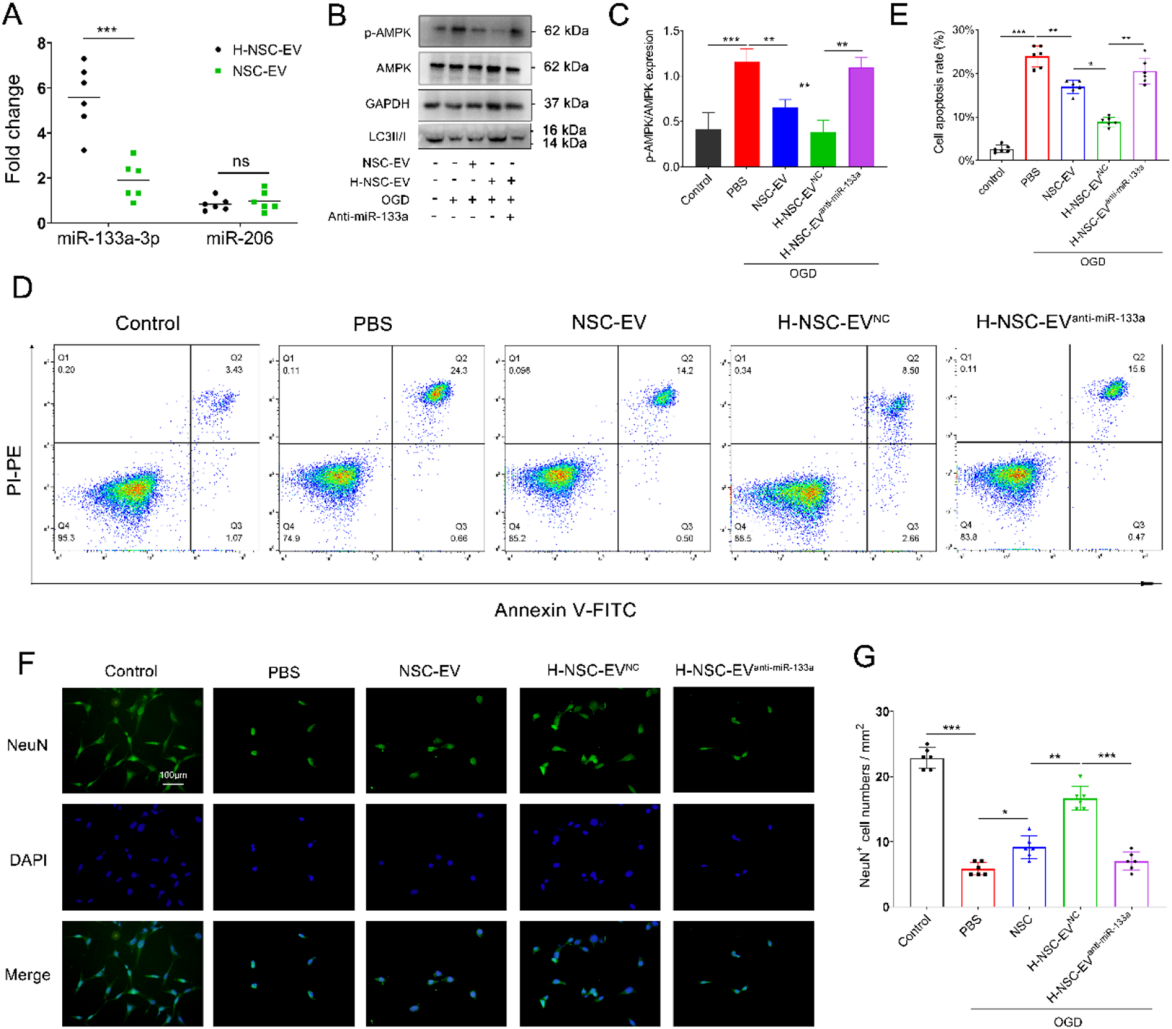
NSC-EVs regulate autophagy and induce neuroprotection by miR-133a-3p . **A** Real-time Quantitative Polymerase chain reaction (qRT-PCR) quantification of miR-133a-3p and miR-206 concentrations in EVs obtained from NSCs pretreated with or without LBP as stated in the materials and methods section. **B–C** Cell apoptosis was examined by flow cytometry in primary neurons exposed to OGD that were treated with PBS, EVs obtained from normal NSCs, EVs isolated from high doses of LBP pretreated NSCs that were pretreated with scramble (H-NSC-EV^NC^) or with anti-miR-133a-3p (H-NSC-EV^anti−miR−133a^). Data are presented as mean ± SD. Neurons were cultured under standard conditions as a control. **F** Representative fluorescent images of OGD-exposed neurons in the treatment group aforementioned (PBS, NSC-EV, H-NSC-EV^NC^, and H-NSC-EV^anti−miR−133a^). **G** The density of NeuN^+^ cells were quantitatively analyzed. Data are statistically different from each other with *P < 0.05, **P < 0.01, and ***P < 0.001

Next-generation sequencing in our previous studies revealed that miR-133a-3P and miR-206 are the main functional molecules of NSC-derived EVs that promote neurological function recovery after stroke [9]. Therefore, we further used qRT-PCR to quantify the enrichment of these two microRNA in the EVs of NSCs treated with LBP. The results showed that LBP intervention increased the enrichment of miR-133a-3p in NSC-derived EVs rather than miR-206 compared with PBS treatment (Fig. 7A). To confirm that the neuronal protection effect of NSC-EV after ischemic stroke was mediated through the expansion of miR-133a-3p, primary neuron cells were co-incubated with PBS, NSC-EV, H-NSC-EV pretreated with anti-miR-133a-3p (H-NSC-EV^anti−miR−133a^), or with H-NSC-EV that had been pretreated with the control oligonucleotide (H-NSC-EV^NC^). After 24 h reoxygenation, LC3-II accumulation and AMPK phosphorylation were significantly lower in the NSC-EV and the H-NSC-EV^NC^ groups than in the PBS group (Fig. 7B–C). However, treatment with H-NSC-EV^anti−miR−133a^ failed to present similar effects on the autophagic flux (Fig. 7B–C).

Consistently, flow analysis revealed that neurons treated with H-NSC-EV^NC^ demonstrated reduced neural apoptosis compared to cells treated with H-NSC-EV^anti−miR−133a^ (Fig. 7D–E). Furthermore, the survival rate of neurons after OGD/R, as assessed by immunofluorescence, was enhanced treated with H-NSC-EV^NC^ when compared to both PBS controls and cells treated with H-NSC-EV^anti−miR−133a^ (Fig. 7F–G). In conclusion, the anti-autophagic activity related to H-NSC-EV administration after OGD/R is partially mediated by increased EV transfer of miR-133a-3p.

## Discussion

Despite ethical concerns and the possibility of immune rejection, NSCs appear ideal for refilling lost neuronal networks. With a deep understanding of such a novel molecule so-called EV, accumulated evidence showed that the benefits of stem cell transplantation are not derived from its direct differentiation capacity but from its ability to secrete bioactive molecules [27–29], which provides a regenerative microenvironment to heal damaged tissue and initiate a self-regulated regenerative response. Our previous studies demonstrated that EVs derived from NSCs promote neuroprotection and neuroregeneration in preclinical stroke models [6, 9]. The efficacy of LBP presented herein in ameliorating stroke outcome suggested that promoting the production of NSC-EVs mediated post-stroke neuroprotection and neurological recovery might be a promising therapeutic strategy for stroke patients. A key mechanism of LBP-induced neuroprotection involves EV-mediated increased delivery of miR-133a-3p from NSCs to the neurons, activating the AMPK/mTOR signaling pathway against autophagic flux.

The presence of various mechanisms makes it complicated to define a single standard therapy for stroke patients. The use of LBP was proven effective in stimulating brain remodeling and ameliorating recovery after stroke, but whether it regulates the EV pathway remains elusive. In the present study, we evaluated the effect of LBP intervention on the characterization of NSC-EV. EVs exhibited similar EV marker expressions without changing the size and purity. And surprisingly, we were able to obtain LBP-pretreated EVs more than 2-fold higher quantity than those collected under standard culture conditions, but not accompanied by damage to the vitality of the source cells, which is contrary to the previously reported methods, including physical and chemical stimulation [30–32]. Therefore, we proposed a feasible method to promote the reproducible generation of NSC-EVs, which may render EVs suitable for disease treatment.

Although a large number of NSC-EVs were obtained after LBP stimulation, this raises the question of the efficacy of these EVs in stroke. As expected, NSC-EVs pretreated with LBP not only retained brain-targeting properties but also presented better neuroprotection and improved rehabilitation ability in stroke than NSC-EVs alone. In addition, we found that LBP-pretreated EV was superior to untreated NSC-EV in improving CBF. Notably, stroke-induced excessive autophagy was further inhibited by LBP-pretreated NSC-EVs, resulting in the expression reduction of Beclin-1 and LC3II, accompanied by an increased abundance of P62. Thus, these results suggested that blocking autophagy at the acute phase of ischemic stroke is beneficial for neurological recovery.

The deterioration of the brain microenvironment caused by the inflammatory cascade after stroke is closely associated with cell death and tissue damage. Consistent with previous studies [33, 34], the dramatic increase in stroke-induced inflammatory cytokines TNF-α, IL-1β, and IL-6 was suppressed after treatment with NSC-EV. Importantly, LBP pre-conditioning NSC-EVs treatment contributed to a more significant inhibitory effect on inflammation than NSC-EVs in a dose-dependent manner. And the inhibition of inflammation level was followed by the reduction of neuronal apoptosis. Here, we proved that LBP can enhance the immunosuppressive effect of NSC-EVs, which may attribute to the interaction between EV and immune cells [35, 36].

Inhibition of autophagy itself does not fully predict the therapeutic influence of such a therapy. On the contrary, the success of this therapeutic intervention depends on the precise autophagic signal pathway that should be blocked. Multiple reports reveal cerebral ischemia induces AMPK phosphorylation inhibiting mTOR [37–39]. The latter is involved in the negative regulation of autophagy by interrupting the interaction of Beclin-1 with lipid kinase VPS34 to subsequently inhibit the nucleation of autophagosomes [40]. In the current study, upregulation of AMPK phosphorylation and downregulation of mTOR phosphorylation were observed, followed by strongly activated autophagy upon induction of OGD. Compared with the effect of NSC-EV treatment in vitro conditions, the application of LBP pre-treated NSC-EVs further reversed the increase of p-AMPK and the reduction of p-mTOR, which resulted in a pronounced downregulation of the autophagy marker LC3-II and contributed to better neuroprotection. Thus, the current study suggests that LBP enhances the inhibitory effect of autophagy mediated by NSC-EVs based treatment of ischemic stroke, although other autophagy-related signaling pathways regulated by NSC-EV cannot be completely excluded. Interestingly, inhibiting the secretion of EVs from NSCs leads to the therapeutic loss of these pretreated EVs, which indicates that EVs, as a member of EV subgroups, plays a vital role in the biological effects observed in this study.

Previous work from our group and other scientists has proved that EVs have pro-angiogenic and anti-inflammatory properties. Intercellular transfer of RNA by small EVs provides a new perspective for exploring the role of these small particles in different physiological systems [41, 42]. To elucidate the underlying mechanism for NSC-EV-mediated neuroprotective and pro-regenerative effects in stroke, we previously performed sequencing to analyze the microRNA profile of NSC-EVs. The results showed that several microRNAs were enriched in EVs, among which we verified that miR-133a-3p and miRNA-206 are responsible for improved neurological function after stroke [9]. Herein, in comparison with NSC-EVs collected under standard culture conditions, LBP pretreatment promoted the enrichment of miR-133a-3p in NSC-EVs, as shown in Fig. 7A, while the enrichment of miR-206 was not significantly different in particles. To further verify that miRNA-133a-3p of NSC-EV cargo is the key cytoprotective compound for neurological recovery by impeding autophagy, NSC-EVs pretreated with anti-miR-25-3p were co-cultured with primary neurons subjected to OGD. As expected, miR-25-3p knockdown eliminated the LBP-promoted therapeutic effect of NSC-EVs, as evidenced by the marked increase in cell apoptosis and loss of neuroprotective ability. Taken together, LBP-pretreated NSC-EV-induced anti-autophagy activity was partly mediated by the activation of the AMPK/ mTOR pathway via the increased delivery of miRNA-133a-3p.

## Conclusion

This study determined that LBP alleviates ischemic brain injury and promotes long-term neurological recovery by modulating the paracrine effects of NSCs. Mechanistically, LBP activated AMPK/mTOR signaling pathway by promoting the enrichment and transfer of miRNA-133a-3p in EVs, thereby inhibiting autophagy activity. Results of the present study clarified the mechanisms of LBP in ameliorating stroke recovery. They supported the application of traditional Chinese medicine Lycium barbarum in the immune regulation of stroke therapy in Asian countries. In addition, the current findings demonstrate the efficacy of targeting production of EVs derived from NSCs to treat stroke, which may be combined with other therapeutic strategies for stroke.

## Data Availability

The raw data supporting the conclusions of this article will be made available by the corresponding author, without undue reservation.
